# Mapping the Research Growth and Citation Impact of Scholarly Works on Sickle Cell Disease in Tanzania: A Scientometric Study

**DOI:** 10.1155/bmri/3018829

**Published:** 2026-08-07

**Authors:** Sydney E. Msonde, Emmanuel C. Balandya, Upendo Masamu

**Affiliations:** ^1^ Directorate of Library Services, Muhimbili University of Health and Allied Sciences, Dar es Salaam, Tanzania, muchs.ac.tz; ^2^ Department of Physiology, Muhimbili University of Health and Allied Sciences, Dar es Salaam, Tanzania, muchs.ac.tz; ^3^ Department of Haematology and Blood Transfusion, Muhimbili University of Health and Allied Sciences, Dar es Salaam, Tanzania, muchs.ac.tz

**Keywords:** scientometric study, sickle cell, sickle cell anemia, sickle cell disease, Tanzania

## Abstract

Sickle cell disease (SCD) remains a major public health concern in Tanzania, where research efforts associated with the Muhimbili Sickle Cell (MSC) Program have contributed to increased scientific output. However, there is limited consolidated evidence on bibliometric trends and research influence in this field. This study examined publication trends, authorship patterns, institutional contributions, and citation‐based research influence of SCD research in Tanzania. A bibliometric analysis was conducted using publications retrieved from Dimensions, Scopus, Web of Science, and Google Scholar for SCD research involving Tanzanian scholars, covering 2003–2024. Records were included if they were peer‐reviewed (articles/reviews) and/or scholarly publications indexed in the selected databases, included at least one author affiliated with a Tanzanian institution, and focused on SCD/hemoglobinopathies or related anemia conditions in Tanzania. English‐language, full‐text, accessible records were included, whereas studies unrelated to Tanzania‐focused SCD or hemoglobinopathies, editorials, opinion pieces, letters, duplicates, records lacking full texts or bibliographic information, or those outside the timeframe were excluded. Google Scholar was used to capture additional records and citation signals that may not be uniformly indexed in other databases. Data were extracted, cleaned, and analyzed using Microsoft Excel, whereas VOSviewer and OriginPro were used for network mapping and visualization. Findings show a progressive/steady increase in SCD research output, with a peak in 2022 and the highest citation accumulation in 2023, indicating growing visibility of recent work. The most productive institutions were MUHAS, NIMR, and CUHAS, and key contributors included Makani J., Nkya S. W., and Sangeda R. Z. High‐impact journals included PLOS One, Malaria Journal, and Blood. The thematic distribution of research output was mainly concentrated in clinical medicine, immunology, and biochemistry. The study demonstrates sustained growth in research visibility and impact in Tanzania, while showing that productivity remains concentrated among a limited number of institutions and authors, highlighting the need for broader participation and thematic diversification.

## 1. Introduction

Research productivity and scientific impact assessment have become increasingly important for understanding the development and contribution of specialized health research areas. Scientometric approaches are widely used to evaluate publication trends, collaboration networks, thematic evolution, institutional productivity, and citation impact across biomedical disciplines [1, 2]. Within this context, sickle cell disease (SCD) research has attracted growing global attention because of the persistent burden of the disease, particularly in sub‐Saharan Africa, where research evidence is essential for informing policy, clinical practice, and disease management strategies.

SCD is one of the most common genetic disorders in Africa [3, 4] and remains a major cause of morbidity and mortality among affected children [5]. Globally, SCD continues to pose significant public health challenges, particularly in low‐ and middle‐income countries where access to early diagnosis, specialized care, and long‐term disease management remains limited. In sub‐Saharan Africa, the disease contributes substantially to childhood mortality, chronic illness, reduced quality of life, and socioeconomic hardship among affected individuals and families [4, 5]. Tanzania is among the five African countries with the highest annual number of newborns affected by SCD (Makani et al., 2018). [3] Although SCD has been incorporated into Tanzania′s noncommunicable disease (NCD) management strategy [6], it continues to impose considerable clinical, social, and economic burdens on individuals, households, and the healthcare system.

Recognizing SCD as a national public health priority, Tanzania established the Muhimbili Sickle Cell (MSC) Program in 2004 to generate country‐specific evidence on disease burden, morbidity, mortality, and clinical management. The program has made substantial contributions to research on the epidemiology, genetics, and clinical manifestations of SCD, including genome‐wide associations between fetal hemoglobin levels and clinical outcomes [6–8]. These efforts have produced an expanding body of scientific publications involving Tanzanian researchers and institutions, reflecting increasing national and international interest in understanding disease mechanisms, improving patient care, and informing evidence‐based policy and practice. SCD research also spans multiple disciplines, including medicine, immunology, genetics, hematology, and biochemistry [6, 8], making it well suited for scientometric assessment of publication patterns, collaboration networks, thematic development, and research influence.

Scientometric analysis has been widely applied in the medical sciences to examine research productivity and scientific development. Previous studies have investigated research trends in infectious diseases such as Ebola [9], broader biomedical research [10], global SCD research [11], SCD research in India [12], SCD research in Nigeria [13], and anaemia‐related research in BRICS countries [14]. Collectively, these studies demonstrate the value of scientometric methods for identifying publication growth, institutional productivity, collaboration patterns, thematic evolution, and scientific influence within specialized health research fields.

Despite the increasing volume of SCD publications in Tanzania, no comprehensive national‐level scientometric study has systematically examined publication trends, authorship patterns, institutional productivity, collaboration networks, thematic evolution, and citation impact of Tanzanian SCD research. Previous bibliometric studies conducted in Tanzania have largely focused on research productivity within specific institutions or disciplines [15], whereas international scientometric studies have examined SCD research globally and in selected countries such as India and Nigeria [11–13]. Consequently, there remains limited consolidated evidence on the evolution, productivity, collaboration patterns, thematic focus, and scholarly impact of SCD research in Tanzania. Addressing this gap is important for strengthening national research strategies, enhancing research visibility, informing funding priorities, and supporting evidence‐based decision‐making for SCD prevention, management, and policy development.

Therefore, this study conducted a scientometric analysis of SCD research in Tanzania to examine publication trends, authorship patterns, institutional productivity, collaboration networks, thematic areas, and citation impact. The findings provide insights into the development and global visibility of SCD research in Tanzania and may support future research planning, funding prioritization, policy development, and the strengthening of collaborative research networks.

## 2. Methodology

### 2.1. Study Design and Data Sources

This study employed a mixed‐methods approach, integrating comprehensive scientometric analysis with qualitative interviews with domain experts to examine research trends, productivity patterns, thematic developments, and major contributors in the field [2] from a Tanzanian perspective. The scientometric component provided quantitative evidence on publication and citation patterns, whereas the qualitative component offered contextual insights into the observed trends and challenges. Findings from both components were integrated during interpretation to provide a comprehensive understanding of the field.

Bibliometric data were retrieved from four major scholarly databases: Dimensions, Scopus, Web of Science, and Google Scholar. These databases were selected to ensure comprehensive coverage of peer‐reviewed literature and grey literature. For example, Dimensions provided advanced filtering tools that aided analysis by enabling exploration of publication trends and citation networks. Scopus facilitated this project′s bibliometric analysis by providing data on publication citation counts and h‐indexes of influential authors, whereas its Scopus Content Discovery (SCD) tools supported the uncovering of emerging research themes. Similarly, the Web of Science provided citation reports to appraise the impact of significant studies and explore interconnected references.

Google Scholar was included as a supplementary database due to its broader coverage of scholarly outputs, particularly in low‐ and middle‐income countries such as Tanzania, where relevant research may appear in institutional repositories, conference proceedings, book chapters, and regional journals not fully indexed in Dimensions, Scopus, or Web of Science databases. This helps reduce publication bias and improve retrieval sensitivity for studies on SCD. Although Google Scholar has limited search reproducibility and lacks controlled indexing, its use was restricted to structured keyword searches, and all records were manually screened based on predefined inclusion criteria (2003–2024; journal articles, conference papers, and book chapters). It was therefore used to complement, not replace, Dimensions, Scopus, and Web of Science.

**Figure 1:**
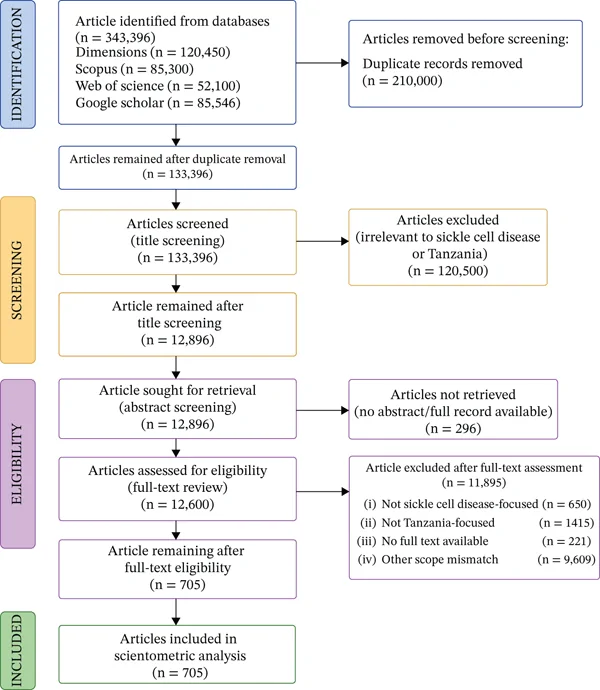
PRISMA Flow Diagram for Article Selection.

A structured search strategy was developed using Boolean operators, phrase searching, and database‐specific field tags. Search terms related to sickle cell disease, sickle cell anemia, sickle cell anaemia, and sickle cell disorder were combined using OR Boolean operator and linked with Tanzania using the AND Boolean operator, and were restricted to publications between 2003 and 2024. Parentheses were applied to ensure the correct logical grouping of concepts. To improve specificity, the standalone term “anemia” was excluded because it retrieved unrelated records. The database‐specific search syntaxes for Dimensions, Scopus, Web of Science, and Google Scholar are presented in Appendix A. [Correction added on “14 August 2026”, after first online publication: In the previous sentence, there was a technical error which made the sentence unclear. The correct text has now been updated in the sentence.]. The searches retrieved 343,396 records, which were imported into EndNote for reference management and duplicate detection. After removing 210,000 duplicate records, 133,396 unique records remained for title screening. Following title screening, 120,500 records unrelated to SCD or Tanzania were excluded, leaving 12,896 records for abstract screening. Of these, 296 records were excluded because the abstract or full text could not be retrieved. The remaining 12,600 articles underwent full‐text eligibility assessment, during which 11,895 articles were excluded because they were not focused on SCD (*n* = 650), were not Tanzania‐focused (*n* = 1415), had no full text available (*n* = 221), or did not meet the study scope (*n* = 9609). Ultimately, 705 publications met the eligibility criteria and were included in the final scientometric analysis (see Figure 1).

#### 2.1.1. Inclusion Criteria

Studies were included if they met all the following conditions:

• Peer‐reviewed journal articles, review articles, conference proceedings, and book chapters
• Articles that had at least one author affiliated with a Tanzanian institution
• Studies focusing on SCD, hemoglobinopathies, or related anaemia conditions in Tanzania
• Publications addressing SCD research productivity, scientometric or bibliometric analysis, or research output trends in Tanzania
• Studies published between 2003 and 2024, corresponding to the establishment period of the MSC Program
• Articles available in the English language
• Full‐text accessible studies indexed in the selected databases

#### 2.1.2. Exclusion Criteria

Studies were excluded if they met any of the following:

• Publications not related to SCD or hemoglobinopathies in Tanzania
• Studies not addressing research productivity, bibliometric/scientometric analysis, or Tanzania‐specific SCD evidence
• Editorials, opinion papers, letters, commentaries, and news articles
• Duplicate records retrieved from multiple (duplicates across) databases
• Studies published outside the timeframe of 2004–2024
• Articles without an accessible full text or insufficient bibliographic information
• Non‐English language publications

Publications that met eligibility criteria were included in the final analysis. English‐language publications were also included to ensure consistency and accurate interpretation of the retrieved records. In addition, grey literature was excluded in order to maintain the quality, uniformity, and comparability of the scientometric dataset. However, these criteria may have introduced language and publication bias by excluding potentially relevant studies published in other languages or disseminated through nonindexed sources. Regarding ethical issues, expert interview participants were provided with informed consent prior to participation. Confidentiality and anonymity were strictly maintained throughout the study, and all data were used exclusively for research purposes.

### 2.2. Data Cleaning and Integrity Assurance

Following a systematic search across Dimensions, Scopus, Web of Science, and Google Scholar, the initial search yielded a global total of 343,396 research outputs for SCD. To ensure the integrity and reliability of the dataset, several data cleaning and validation procedures were undertaken. First, duplicate records across databases were identified and removed. Second, all records were screened for relevance based on titles, abstracts, and keywords to exclude unrelated publications. Third, metadata fields such as author names, institutional affiliations, and publication years were standardized to address inconsistencies across databases. Fourth, incomplete and erroneous records were excluded. Finally, cross‐validation of records across Dimensions, Scopus, Web of Science, and Google Scholar was conducted to ensure the dataset′s consistency and accuracy. After narrowing the search to include only research outputs affiliated with Tanzania, a total of 705 publications were returned for the period between 2003 and 2024.

### 2.3. Scientometric Data Analysis

**Figure 2:**
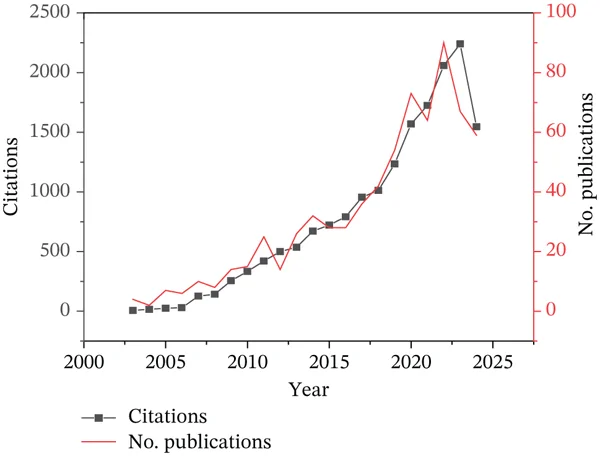
Year‐wise publication and citation trends.

**Figure 3:**
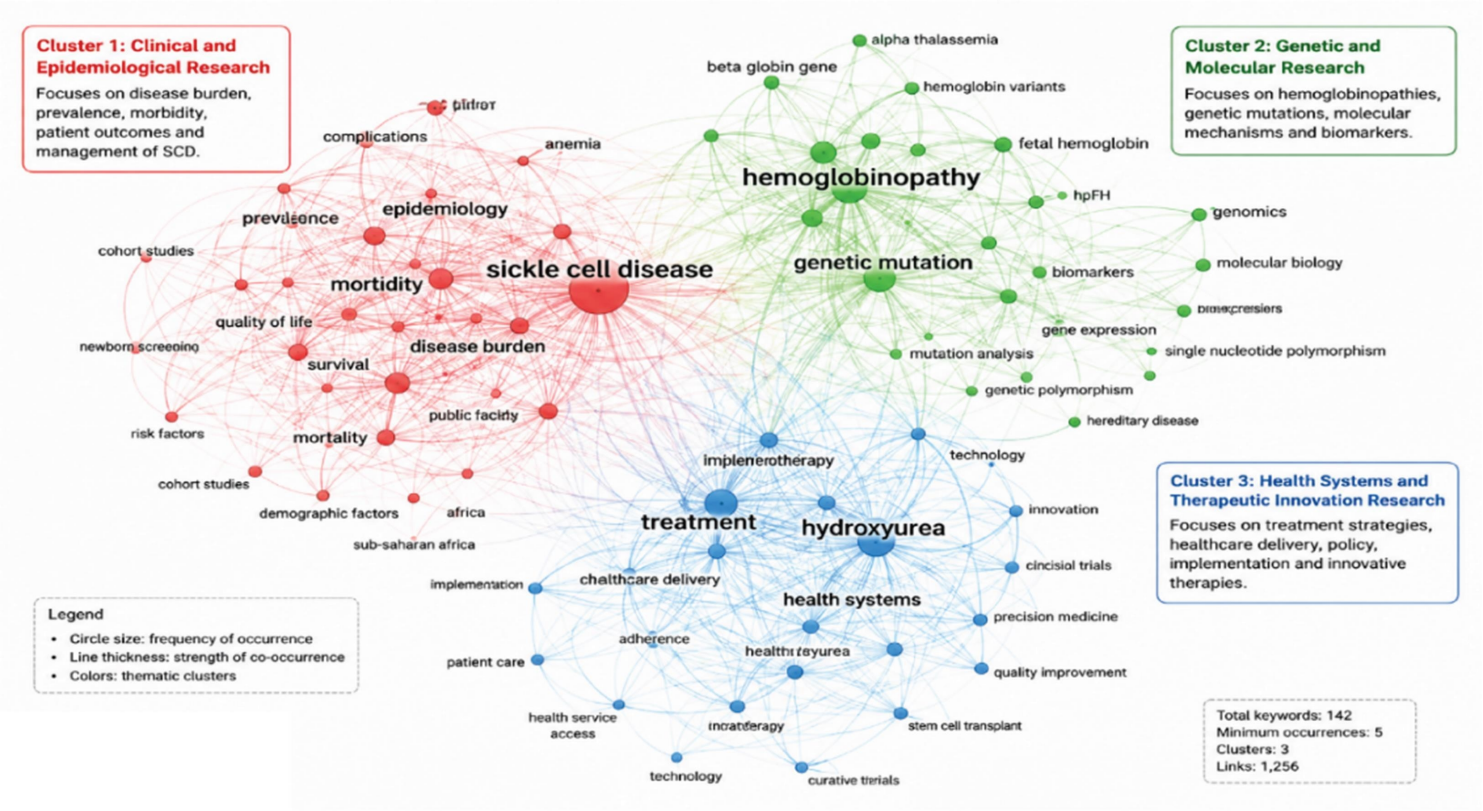
Keyword co‐occurrence network of sickle cell disease research.

**Figure 4:**
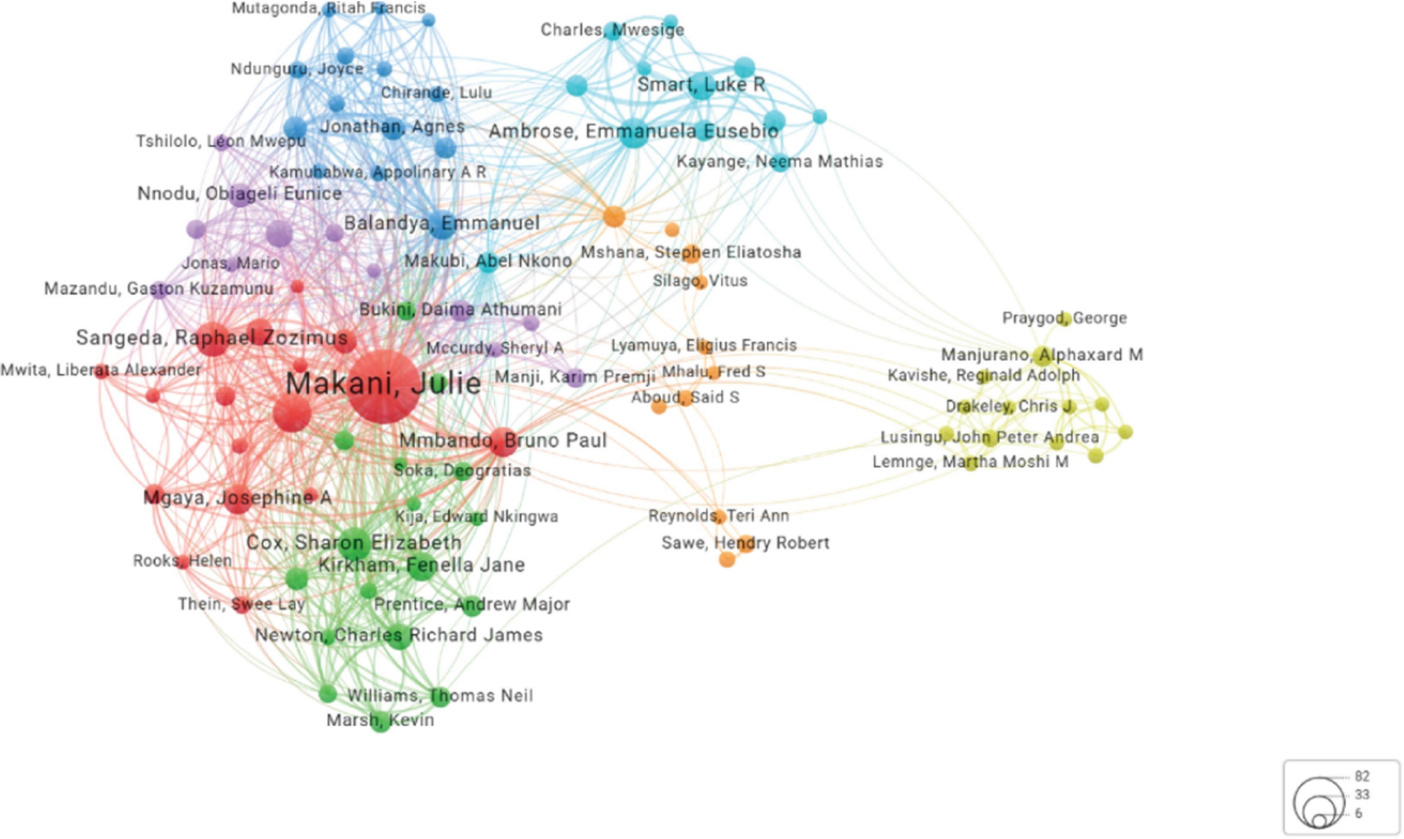
Visualization of the author′s research productivity.

The cleaned data were analyzed using established bibliometric techniques, including publication and citation analysis, co‐authorship analysis, institutional productivity, and key subject analysis, among others, employing methodological frameworks in bibliometric research [16], as well as descriptive statistics. Microsoft Excel and OriginPro were used to generate graphical representations of annual publication trends in SCD research in Tanzania over the 20‐year period, highlighting growth patterns and key peaks in research activity. Similarly, bibliometric network visualization and mapping were performed using VOSviewer (Version 1.6.20) to identify and visualize relationships among authors, institutions, countries, keywords, and cited references [17]. Bibliographic records retrieved from the Dimensions database for the period 2003–2024 were imported into the software to analyze author publication and citation trends (Figure 2), keyword co‐occurrence (Figure 3), and authors research productivity (Figure 4). A minimum threshold of two publications per unit was applied to optimize network clarity. Relationships between items were calculated using the association strength normalization method, whereas clusters were generated using VOSviewer′s default modularity‐based clustering algorithm. In the network maps, node size represents the volume of publications or the relative weight of an item, line thickness indicates the strength of co‐authorship or citation links, and colors denote distinct collaborative or thematic clusters.

To triangulate the quantitative bibliometric findings, eight purposively selected domain experts with demonstrated experience in SCD research were interviewed. Selection criteria included professional experience, research involvement, and publication history in the SCD field. A semistructured interview guide was developed to explore participants′ perspectives on research trends, challenges, opportunities, and future directions in SCD research. Interviews were conducted virtually via Zoom and continued until thematic saturation was reached. Each interview lasted approximately 15 min and was audio‐recorded with participants′ consent before being transcribed verbatim.

The interview transcripts were analyzed using an inductive thematic analysis approach following the procedures outlined by [18]. Initially, the transcripts were read repeatedly to achieve data familiarization. Open coding was then performed to identify meaningful segments of text relevant to the study objectives. Similar codes were subsequently grouped into categories, from which broader themes were developed and refined through iterative review and comparison across transcripts. Coding and theme development were undertaken independently by two members of the research team, and discrepancies were discussed until consensus was reached. To enhance the credibility and trustworthiness of the findings, peer debriefing and cross‐checking of codes and themes were conducted among the researchers. The resulting themes were then integrated with the bibliometric findings to provide a comprehensive understanding of SCD research trends, collaboration patterns, challenges, and emerging research priorities in Tanzania.

## 3. Results

The indicators analyzed based on the study objectives were several publications and citation growth, productive authors, and productive institutions based on several publications and citation counts, which were normalized by publication year to account for time‐dependent accumulation. To ensure a clear distinction between quantitative and qualitative evidence, the findings from the scientometric analysis and the expert interviews are presented separately in the subsequent sections.

### 3.1. Quantitative Findings from the Scientometric Analysis

#### 3.1.1. Publication and Citation Growth

The bibliometric analysis of 705 SCD publications retrieved from Dimensions, Scopus, Web of Science, and Google Scholar indicates a consistent upward trend in research output from 2003 to 2024, with notable peaks in 2011, 2014, 2020, and 2022, as shown in Table 1.

**Table 1:** Year‐wise publication and citation trends.

*Item*		Publication year																					
	2003	2004	2004	2006	2007	2008	2009	2010	2011	2012	2013	2014	2015	2016	2017	2018	2019	2020	2021	2022	2023	2024	Total
Published article	4	2	7	6	10	8	14	15	25	14	26	32	28	28	36	43	54	73	64	90	68	59	705
Total citation	7	17	25	31	127	143	256	334	421	500	537	671	723	792	956	1013	1235	1569	1725	2059	2241	1547	18,323

Citation patterns similarly show an overall increase over time, with exponential growth after 2007 and a decline observed in 2024, as shown in Figure 2.

Bibliometric mapping using VOSviewer keyword co‐occurrence analysis identified three major thematic clusters in SCD research, as illustrated in Figure 3. The first cluster focused on clinical and epidemiological studies of disease burden, prevalence, morbidity, and patient outcomes. The second cluster comprised genetic and molecular research on hemoglobinopathies, genomic mechanisms, and biomarkers.

The third cluster encompassed health systems, treatment strategies, and therapeutic innovations, including hydroxyurea use, healthcare delivery, and emerging gene‐based therapies.

#### 3.1.2. Top 15 Productive Authors

The bibliometric analysis of 705 SCD publications depicted in Table 2 shows that research productivity is concentrated among a relatively small group of authors, with Makani, J. (Np 184),^1^ Nkya, S. W. (Np = 49), and Sangeda, R. Z. (Np = 40) emerging as the most productive, all affiliated with Muhimbili University of Health and Allied Sciences (MUHAS), alongside other contributors such as Emmanuela E. Ambrose (Np = 30) from CUHAS. Citation analysis indicates that authors such as Makani, J. (h‐index 35; Tc, 3705)^2^ and Luzzatto, L. (Tc = 808; h‐index = 50) exhibit high citation impact despite differences in publication volume, suggesting that research visibility, novelty [19], and collaboration patterns [20] play an important role in determining scholarly influence.

**Table 2:** Top 15 productive authors.

S/N	Name of researchers	Affiliation	Publication and citation impact			
			*Publications frequency*	*Citations trends*	*Citation mean*	*h-index*
1	Julie Makani	MUHAS	191	3,710	19.42	35
2	Siana W. Nkya	MUHAS	54	475	8.80	14
3	Raphael Z. Sangeda	MUHAS	44	261	5.93	17
4	Emmanuela E. Ambrose	CUHAS	30	321	10.70	13
5	Bruno B. Mmbando	NIMR	30	441	14.70	32
6	Emmanuel C. Balandya	MUHAS	29	210	7.24	11
7	Josephine A. Mgaya	MUHAS	28	788	28.14	12
8	Lucio Luzzatto	MUHAS	26	808	31.08	50
9	Alphaxard M. Manjurano	NIMR	21	918	43.71	28
10	Upendo Masamu	MUHAS	19	41	2.16	5
11	Agnes Jonathan	MUHAS	19	66	3.47	8
12	Irene K. Minja	MUHAS	19	89	4.68	11
13	Salim M. K. Abdulla	IHIT	18	718	39.89	56
14	Daima A. Bukini	MUHAS	18	70	3.89	6
15	Paschal J. Ruggajo	MUHAS	17	73	4.29	11

Further analysis using VOSviewer reveals seven co‐authorship clusters that reflect distinct but interconnected thematic and collaborative groupings within SCD research (see Figure 4). These clusters can be interpreted as representing clinical and translational hematology, epidemiology and public health research, genetics and molecular studies, parasitology and infectious disease interactions, health systems and implementation research, clinical outcomes and pediatric health, and multidisciplinary regional collaborations.

#### 3.1.3. Top 10 Productive Institutions

A total of 69 institutions were involved in conducting research on “SCD” from a Tanzanian perspective. The bibliometric analysis identified “institutional productivity and citation impact” as the primary themes for assessing research performance. Institutional research productivity was measured by the number of research articles published and the citation scores received. Table 3 presents the top 10 most productive institutions in Tanzania. The MUHAS (Np, 342; Tc, 8228) ranked first in SCD research productivity, followed by the National Institute for Medical Research (Np, 87; Tc, 2494) and the Catholic University of Health and Allied Sciences (Np, 73; Tc, 1005), among others. These institutional citation scores were based on the number of SCD papers published by scholars at each institution. The findings further indicate a theme of “institutional concentration in research output”, with a relatively small number of institutions accounting for a large share of both publications and citations per year.

**Table 3:** Top 10 institutions by scholarly output.

	Institution name	No. of publications	Citation score	Citation mean
1	Muhimbili University of Health and Allied Sciences	342	8228	24.06
2	National Institute for Medical Research	87	2494	28.67
3	Catholic University of Health and Allied Sciences	73	1005	13.77
4	Muhimbili National Hospital	57	825	14.47
5	Tumaini University (TUMA)	48	1945	40.52
6	Bugando Medical Centre	42	562	13.38
7	Ifakara Health Institute	42	1378	32.81
8	University of Dar es Salaam	42	606	14.43
9	Kilimanjaro Christian Medical University College	36	2621	72.81
10	University of Dodoma	28	99	3.54

**Figure 5:**
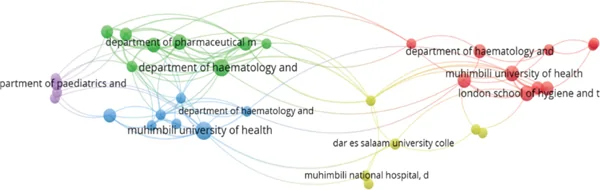
Institution co‐authorship.

Conversely, the institutional research collaboration network generated in VOSviewer (see Figure 5) revealed a third key theme: “collaborative clustering among institutions and departments,” reflecting structured patterns of institutional cooperation in SCD research. The links between institutions and their associated departments are shown with colored lines, whereas the sizes of the circles indicate the number of articles produced by scholars at each institution. The network visualization map showed five clusters, each representing distinct collaboration groupings that reflect both national and international research linkages and varying levels of institutional centrality.

Cluster 1 (red) comprised 10 institutions, including the MUHAS, the London School of Hygiene and Tropical Medicine (United Kingdom), and Muhimbili National Hospital. Cluster 2 (green) included eight MUHAS‐related departments, such as the Department of Haematology and Blood Transfusion, the Department of Biochemistry, and the MSC Program. Similarly, Cluster 3 (pale blue) consisted of seven institutions, including the MUHAS, the Sickle Cell Foundation of Ghana, and the Centre of Excellence for Sickle Cell Disease and Training in Nigeria. Cluster 4 (yellow) included six institutions, such as the National Institute for Medical Research, Muhimbili National Hospital, and Dar es Salaam University College of Education. Finally, Cluster 5 (purple) comprised four institutions, including Bugando Medical Centre, the Catholic University of Health and Allied Sciences, and the Division of Haematology at Cincinnati Children′s Hospital Medical Centre, United States.

#### 3.1.4. Medium of Research Communication

Based on data retrieved from the different databases, a total of 705 research outputs on SCD were published by Tanzanian scholars. The majority were journal articles (623; 88.3%), followed by preprints (66; 9.3%), book chapters (14; 2%), whereas conference papers and monographs were minimally represented (one each; 0.2%). These patterns may indicate a strong dominance of journal‐based scholarly communication in SCD research.

#### 3.1.5. Most Productive and Impactful Journals

The bibliometric analysis of 160 journals contributing to SCD research based on merged datasets from Dimensions, Scopus, Web of Science, and Google Scholar, and following data cleaning, including duplicate removal, shows that publication output is concentrated in a relatively small number of high‐impact journals. The top 15 journals accounted for 185 publications, with leading outlets including PLOS One (Np = 27), Blood (Np = 22), and Malaria Journal (Np = 22). High‐impact journals such as The Lancet and Clinical Infectious Diseases (see Table 4), despite publishing fewer SCD‐related articles, demonstrated disproportionately high citation counts (e.g., The Lancet Tc = 843; Clinical Infectious Diseases Tc = 585), indicating that fewer but highly visible publications generate substantial scholarly influence (Stevens et al., 2022) [21].

**Table 4:** Journal publication output.

S/N	Journal name	No. of publications	Total citation	Citation mean	h‐index
1.	PLOS One	27	1014	37.59	435
2.	Malaria Journal	22	586	26.64	120
3.	Blood	22	597	27.14	525
4.	British Journal of Haematology	15	345	23.00	208
5.	Tanzania Journal of Health Research	13	124	9.54	30
6.	BMJ Open	11	61	5.55	160
7.	American Journal of Hematology	11	146	13.27	130
8.	The Lancet Haematology	10	178	17.80	93
9.	American Journal of Tropical Medicine and Hygiene	9	479	53.22	169
10.	The Journal of Infectious Diseases	9	471	52.33	276
11.	Frontiers in Genetics	8	60	7.50	120
12.	The Lancet	8	843	105.38	895
13.	Clinical Infectious Disease	8	585	73.13	387
14.	BMC Pediatrics	6	72	12.00	100
15.	BMC Public Health	6	138	23.00	197
		185	5699	30.80	

**Figure 6:**
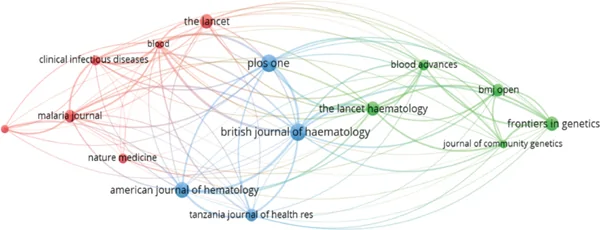
Productive and impactful journals.

Three main thematic journal clusters were identified using network visualization in VOSviewer (see Figure 6). The first cluster represents “high‐impact clinical and biomedical journals” such as Blood, The Lancet, and Clinical Infectious Diseases, which are strongly connected through high citation linkages. The second cluster comprises “specialized hematology and genetics journals,” including Blood Advances and Lancet Haematology, focusing on disease mechanisms and advanced therapeutic research. The third cluster includes “broad‐access and regional health journals” such as PLOS One, the British Journal of Haematology, and the Tanzania Journal of Health Research, reflecting a combination of global accessibility and contextual relevance.

#### 3.1.6. The Most Published Subject Areas

**Figure 7:**
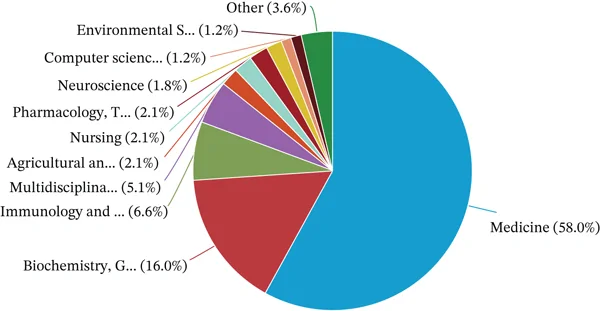
Most published SCD research areas.

The bibliometric analysis of 705 SCD publications retrieved from the Dimensions database shows that research is concentrated in a few dominant subject areas. Figure 7 shows that medicine accounted for the largest share (Np = 408; 58%), followed by biochemistry, genetics, and molecular biology (Np = 113; 16%), immunology and microbiology (Np = 46; 6.6%), and multidisciplinary research (Np = 36; 5.1%). The observed pattern may reflect a strong biomedical orientation in SCD research.

### 3.2. Qualitative Findings From Domain Expert Interviews

#### 3.2.1. Perceptions of Research Trends

In exploring the relationship between publication and citation growth, we interviewed domain experts to reflect on how publication trends have evolved in their field and to identify the factors that most affect citation rates for their articles. The qualitative analysis of expert interviews generated four interrelated themes that explain and contextualize the bibliometric patterns. The first theme concerns *a publish-or-perish academic culture*, in which publication output is increasingly tied to institutional recognition and performance metrics. One participant noted that

“Because we′re so focused on things like how many times a paper is cited or someone′s h‐index, there′s a bigger push to just publish more stuff, and to do it on purpose.” (DE_1_)^3^

The second theme concerns *strategic publishing and citation maximization*, where researchers intentionally select topics and journals likely to attract citations. This is reflected in the statement provided by one of the domain experts:

“There′s a trend towards more visibility—people are choosing topics they feel may get them cited, which wasn′t something we saw a decade ago.” (DE_4_)

The third theme focuses on *journal prestige, indexing, and dissemination channels as determinants of citation impact.* The domain experts emphasized that publishing in well‐known, well‐indexed journals significantly increases visibility and the likelihood of citations per year, reinforcing the importance of journal selection strategies in scholarly communication. The fourth theme highlights *alignment with global health priorities,* particularly Sustainable Development Goal 3 (Good Health and Well‐being), as noted by one domain expert through an interview.

“I think the trend will shift towards global health‐related issues… researchers in these areas are more likely to have papers that receive higher citation scores” (DE_2_)

The interview data provide explanatory depth to the SCD research trends. The observed growth in publications and concentration in high‐impact thematic areas can be understood in light of the publish‐or‐perish culture and citation‐driven publishing strategies described by participants, as summarized by one domain expert who noted that “success and institutional recognition often depend on how many you publish, ” illustrating how academic incentives shape research output.

#### 3.2.2. Perceptions of Productive Authors

To complement the bibliometric findings, interviews with domain experts revealed four interrelated themes that explain patterns for keeping them productive during the research process. The first theme identifies intrinsic and institutional motivations for productivity, with participants linking academic success and recognition to high publication volume, as illustrated by the view that “success and institutional recognition often depend on how many you publish.”

The second theme concerns strategic topic selection for visibility and citation potential, with participants noting that “researchers are choosing topics they feel may get them cited” (DE_3_), reflecting a deliberate orientation toward high‐impact research areas. The third theme highlights collaboration as a key driver of productivity, with one interviewee explaining that “most of my publications come through collaborative projects (DE_4_), it distributes the workload and opens doors to higher‐impact journals,” underscoring the role of co‐authorship in enhancing both output and quality. The fourth emphasizes a focus on policy‐relevant, timely research, with participants stating that targeting socially and policy‐relevant topics increases publication opportunities and citation impact.

#### 3.2.3. Perceptions of Research Communication and Journal Impact

Regarding the distribution of document types, two key themes emerged from the expert interviews. The first theme is an institutional and career‐driven preference for journal publications, where journal articles serve as the primary currency for academic recognition, promotion, and funding. These views are exemplified by one of the participants who noted that:

“I tend to favour journal articles as they tend to hold more academic value, and are more likely to be considered by colleges for promotion and grant applications.” (DE_3_)

The second theme is selective, opportunity‐driven publishing in alternative formats, particularly book chapters and conference outputs. Interviewees indicated that book chapters are typically produced in response to specific invitations, as reflected in the statement:

“When invited, I sometimes write book chapters, especially if I know an established editor will be involved in the book.” (DE_6_)

On the other hand, conference papers and monographs were described by participants as less attractive due to limited institutional recognition, time constraints, and weaker institutional rewards. Regarding journal selection and perceived impact, three themes emerged. The first theme is journal prestige and global visibility, with participants preferring widely indexed international journals for their global reach. This view was noted in one of the interviewees, who noted that:

“I view PLOS One and The Lancet as having a broad reach with respect to attracting a lot of attention from scientists, doctors, and the general public all over the world.” (DE_8_)

The second theme addresses functional impact beyond bibliometric indicators, describing journal influence in relation to scientific and policy needs rather than to metrics alone. As one domain expert explained,

“To my understanding, a journal′s influence is not measured solely by numerical indices… but by how effectively it addresses urgent local, regional, and global health challenges.” (DE_5_)

The third theme concerns the contextual and policy relevance of regional journals, where Tanzanian journals were valued for their direct applicability to local health systems. One participant noted that:

“I perceive the Tanzania Journal of Health Research… speaks to our context and is appealing to policymakers,” DE_7_

#### 3.2.4. Perceptions on the Most Published Research Area

When asked to explain the subject area they consider most relevant to advancing research in SCD, the domain experts identified two subject areas and provided justifications for their choices. The first theme is biomedical dominance in SCD research, with participants identifying medicine, biochemistry, and molecular biology as the core disciplines. Interviewees emphasized that these fields are central to understanding disease mechanisms, improving clinical outcomes, and addressing childhood morbidity and mortality.

The second theme is capacity building and the prioritization of future research, with experts emphasizing the need to strengthen laboratory research, clinical studies, and interdisciplinary approaches through sustained investment in infrastructure, training, and funding. They noted these variables as essential to advance diagnostic and therapeutic innovations.

## 4. Discussion

SCD remains a major public health problem globally because of its high mortality, debilitating complications [22], and profound social consequences [23]. Tanzania′s establishment of the MSC Program in 2004 therefore represented an important strategic investment in generating locally relevant evidence to improve clinical care and public health responses [6]. The progressive increase in Tanzanian SCD publications observed in this study suggests that this investment has gradually strengthened national research capacity rather than simply increasing publication numbers. The marked growth after 2010 likely reflects the maturation of long‐term research programs, expanded international collaborations, and sustained investment in SCD research aimed at understanding disease severity and improving patient management [13]. Similar upward publication trends have also been reported globally, reflecting increasing scientific attention to SCD over the last decade [4].

Although citation counts increased steadily and peaked in 2023, these findings should be interpreted cautiously. Citation indicators primarily reflect scholarly visibility, knowledge dissemination, and scientific influence rather than direct improvements in clinical care or patient outcomes [24]. The subsequent decline in citations is therefore more plausibly explained by the shorter citation window available for recently published articles than by a decline in research quality. Because citations accumulate over time, recently published papers have had fewer opportunities to be cited. Consequently, citation growth should be interpreted as evidence of increasing international recognition of Tanzanian SCD research rather than a direct measure of clinical effectiveness [4, 24]. It should be noted that both raw citation counts and normalized citation rates (citations per document and per year) were used to provide complementary perspectives on research impact and temporal trends.

As such, the observed average research productivity of 1.1 documents per author per year and 1.2 citations per document per year suggests that, despite sustained publication growth, Tanzanian SCD research remains concentrated within a relatively small group of highly active researchers. Compared broadly with other high‐burden African settings such as Nigeria and Ghana [13], this pattern indicates that opportunities remain to broaden national research participation and strengthen research capacity across institutions. The qualitative findings provide additional insight into this pattern by showing that publication decisions are strongly shaped by institutional promotion systems and research funding expectations, where journal articles are regarded as the principal measure of academic performance. This institutional incentive structure encourages publication in internationally visible journals, thereby enhancing research visibility and citation performance [12]. At the same time, participants emphasized that regional journals continue to play an important role by disseminating context‐specific evidence that directly informs national policy and clinical practice, despite receiving relatively fewer citations [11]. These findings suggest that research influence should be evaluated from both international scholarly visibility and local health‐system relevance rather than citation metrics alone [11–13].

The concentration of highly productive authors within MUHAS, complemented by contributions from CUHAS and NIMR, illustrates the central role that a relatively small number of institutions play in driving SCD research in Tanzania. Such institutional concentration is common in many low‐ and middle‐income countries, where specialized centers possess stronger research infrastructure, sustained funding opportunities, and broader international collaborations. Interestingly, authors such as Alphaxard M. Manjurano and Salim M. K. Abdulla achieved comparatively high citation influence despite publishing fewer articles, demonstrating that publication volume alone does not necessarily determine scholarly impact [24]. Rather, methodological rigor, scientific relevance, innovation, and the quality of collaborative research often determine whether studies become widely cited within the international scientific community [21]. These findings suggest that strengthening research capacity across additional Tanzanian institutions may broaden national expertise while preserving the high‐quality outputs currently generated by established research centers.

The extensive co‐authorship network observed among Tanzanian and international researchers demonstrates that collaboration has become a fundamental mechanism for advancing SCD research in Tanzania. Beyond increasing publication output, collaborative networks facilitate multidisciplinary expertise, mentorship, access to specialized laboratory facilities, and international funding opportunities, thereby strengthening research quality. These findings support previous evidence that national and international collaborations substantially improve research productivity and scientific influence [25]. The qualitative findings further reinforce this interpretation by indicating that collaborative partnerships are deliberately cultivated to increase publication opportunities, expand research visibility, and strengthen institutional capacity. Consequently, the collaboration patterns identified in this study provide important insights into the structural organization of SCD research in Tanzania [4]. They demonstrate how institutions such as MUHAS, NIMR, and CUHAS function as interconnected centers of scientific excellence that collectively sustain national research capacity.

The predominance of journal articles reflects more than researchers′ preference for a particular publication format. The qualitative findings indicate that journal publications are strongly incentivized within academic institutions because they constitute the principal evidence for promotion, funding applications, academic recognition, and career advancement. This explains why conference papers, books, and monographs are comparatively underrepresented despite their value for scholarly communication. The bibliometric findings further show that researchers preferentially publish in international journals to maximize global visibility, citation potential, and opportunities for international collaboration [26, 27]. Moreover, journals remain the preferred communication channel because of the perceived rigor of peer review and the credibility associated with publishing high‐quality scientific work [13]. Although publication in journals with higher h‐indices generally enhances citation performance [28], the qualitative findings reveal an equally important dimension that is not captured by bibliometric indicators alone. Participants emphasized that regional journals remain highly valuable because they disseminate contextually relevant evidence that directly informs national policy and clinical practice. Taken together, these findings indicate that journal impact in Tanzanian SCD research is shaped by two complementary dimensions: international scholarly visibility reflected by citation metrics and local health‐system relevance reflected by policy and practice influence [26–28].

This study found a predominance of research in medicine, biochemistry, immunology, and blood transfusion. This pattern reflects a strong emphasis on improving the diagnosis, clinical management, and survival of individuals living with SCD in Tanzania. Previous studies have demonstrated the importance of newborn screening programs, fetal haemoglobin genetics, alpha‐thalassemia, glucose‐6‐phosphate dehydrogenase (G6PD) deficiency screening, and transfusion medicine in improving patient outcomes [29, 30]. The qualitative findings complement these bibliometric results by demonstrating that researchers intentionally prioritize biomedical investigations because these disciplines are perceived to offer the greatest potential for improving clinical management and patient survival. In addition, participants recognized the growing need for interdisciplinary research incorporating behavioral science, implementation science, health systems research, and health economics to address persistent barriers to access and quality care [31]. These findings suggest that while biomedical research appropriately dominates the current evidence base, expanding interdisciplinary approaches could further strengthen Tanzania′s response to the complex healthcare needs associated with SCD [30, 31].

Importantly, this research highlights the convergence of the bibliometric and qualitative findings, which demonstrates how collaborative research networks have facilitated the translation of scientific evidence into national health policy and clinical practice. Rather than functioning solely as academic partnerships, these collaborations have supported large‐scale initiatives such as the Sickle Cell Access and Lifelong Care Program (SCALE), the partnership between the Government of Tanzania and Novartis through the Africa Sickle Cell Disease Program, the Sicklecare Project, and the continued expansion of the MSC Program. The qualitative findings indicate that these partnerships are valued not only for increasing scientific productivity but also for strengthening evidence‐informed decision‐making and improving healthcare delivery. Consequently, the collaborative structures identified in this study illustrate how sustained academic partnerships can extend beyond knowledge production to support health‐system strengthening, workforce development, and long‐term policy implementation.

Thus, this study highlights several critical perspectives. First, it underscores Tanzania′s expanding public health awareness regarding SCD as a major driver of regional morbidity and mortality. Second, the steady upward trajectory of research productivity reflects improving collaborative depth and academic capacity. Third, the mapped institutional and authorship networks offer transparent evidence of strategic partnerships that can be leveraged to guide future policy actions, optimize health system funding, bridge localized knowledge gaps [30, 32], and contextualize Tanzania′s research contributions within the broader global scientific landscape.

## 5. Study Limitations

Although this study provides valuable insights into SCD research in Tanzania, several limitations should be acknowledged. First, despite using multiple databases, variations in coverage and indexing policies across Dimensions, Scopus, Web of Science, and Google Scholar may have led to the omission of some relevant publications, potentially affecting the dataset′s completeness. Second, the exclusion of grey literature, such as theses, institutional reports, and locally published documents, may have introduced publication bias and limited the inclusion of context‐specific evidence not indexed in major databases.

Third, although the study provides a national‐level overview of Tanzania, it does not capture potential regional or institutional variations in research productivity, which may be important for understanding localized research capacity and disease burden dynamics. Fourth, the observed decline in citations after 2023 may partly reflect the short citation window and the time required for newly published studies to gain visibility and accumulate citations, rather than a true decline in research influence.

Fifth, citation counts were analyzed in their raw form; therefore, the influence of publication age may affect citation accumulation. This limitation is acknowledged, as older publications tend to accumulate more citations than recent ones. Sixth, the scientometric approach used in this study is inherently quantitative and does not assess the clinical relevance or real‐world application of research findings, thereby providing only a partial view of research influence. Finally, the temporal scope of the analysis may limit the interpretation of long‐term trends, particularly regarding emerging therapies and evolving management strategies for SCD.

## 6. Conclusions

This study mapped the SCD research landscape in Tanzania using bibliometric methods, identifying 705 publications produced, demonstrating a steady increase in research productivity over the past two decades. The findings show that research activity in this area is concentrated within a small number of institutions, particularly MUHAS, NIMR, and CUHAS, with strong reliance on international journal publications and sustained cross‐institutional collaboration.

Thus, these results indicate increasing scientific productivity and international visibility of Tanzanian SCD research, although output and influence remain unevenly distributed across institutions and researchers. The findings provide a clear baseline for understanding the structure and evolution of SCD research in Tanzania and its positioning within the global research landscape.

The study is limited by its reliance on indexed bibliometric databases, which may exclude nonindexed publications and grey literature, and by the use of citation‐based indicators that do not directly measure clinical or health system impact. Despite these limitations, the findings provide a structured overview of research development patterns in this field.

## Author Contributions

Sydney E. Msonde conceptualized the study, developed the methodology, undertook data collection and bibliometric analysis, supervised the research process, and led the manuscript writing. Emmanuel C. Balandya contributed to the interpretation of bibliometric results and provided critical revision of the manuscript. Upendo Masamu assisted with data curation, visualization, and literature review.

## Funding

Emmanuel Balandya and Upendo Masamu are supported by the Sickle Pan‐African Research Consortium (SPARCO) Clinical Coordinating Center (SPARCO–CCC; U24 HL135881) and SPARCO‐Tanzania (U01 HL156853), respectively, funded by the National Heart, Lung, and Blood Institute (NHLBI) of the National Institutes of Health (NIH). The content of this paper is solely the responsibility of the authors and does not necessarily reflect the official views of the NIH. Emmanuel Balandya and Upendo Masamu are supported by the Sickle Pan‐African Research Consortium (SPARCO)–Tanzania U01 HL156853.

## Disclosure

All authors read and approved the final version of the manuscript.

## Conflicts of Interest

The authors declare no conflicts of interest.

## Endnotes

^1^The number enclosed in brackets (e.g., Np, 71) indicates total articles an institution or author published over the period of 20 years. Np, implies Number of Publications.

^2^The number enclosed in brackets (e.g., h‐index, 54; Tc, 1232) indicates an author h‐index and total citations an article received over the period of 20 years.

^3^The letters enclosed in brackets (e.g., DE_1,_ DE_2…_) indicate the quotation number of the *Domain Expert* interviewed.

## Data Availability

The data that support the findings of this study are available from the corresponding author upon reasonable request.

## Appendix A: Database‐Specific Search Syntaxes

1. Search Strategy Used in the Dimensions Database

Search Database: Dimensions (Publications).

Search Fields: Title and Abstract.

Search Query:

(“sickle cell disease” OR “sickle cell anemia” OR “sickle cell anaemia” OR “sickle cell disorder”) AND (Tanzania OR Tanzania ^∗^)

Filters:

Publication Year = 2003–2024.

Publication Type = Article OR Conference Proceeding OR Book Chapter.

2. Search Strategy Used in the Scopus Database

Search Database: Scopus.

Search Fields:

TITLE‐ABS‐KEY.

Search Query: (TITLE‐ABS‐KEY (“Sickle Cell Disease”) OR TITLE‐ABS‐KEY (“Sickle Cell Anemia”) OR TITLE‐ABS‐KEY (“Sickle Cell Anaemia”) OR TITLE‐ABS‐KEY (“Sickle Cell Disorder”)) AND (LIMIT‐TO (DOCTYPE, “ar”) OR LIMIT‐TO (DOCTYPE, “cp”) OR LIMIT‐TO (DOCTYPE, “ch”)) AND (LIMIT‐TO (LANGUAGE, “English”)) AND (LIMIT‐TO (AFFILCOUNTRY, “Tanzania”)) AND PUBYEAR >2003 AND PUBYEAR <2024.

3. Search Strategy Used in the Web of Science Database

Search Database: Web of Science.

Search Query: TS = (“Sickle Cell Disease” OR “Sickle Cell Anemia” OR “Sickle Cell Anaemia” OR “Sickle Cell Disorder”) AND DT = (Article OR “Proceedings Paper” OR Book Chapter) AND LA = (English) AND CU = (Tanzania) AND PY = (2004‐2023).

4. Search Strategy Used in the Google Scholar Database

Search Database: Google Scholar.

Search Field: Advanced Search.

Search Query (with all of the words): (“sickle cell disease” OR “sickle cell anemia” OR “sickle cell anaemia” OR “sickle cell disorder”) AND Tanzania.

Return articles dated between 2003 and 2024 (or using Google Scholar′s “custom range” tool).

*Note:* Google Scholar was included to enhance coverage of grey literature and early‐stage publications not indexed in traditional databases. Due to its limited advanced search functionality, results were manually screened and filtered according to predefined inclusion criteria.
